# 4D Flow MRI at 0.6 T—Self-Gating Versus Camera-Based Respiratory Binning

**DOI:** 10.3390/bioengineering13030282

**Published:** 2026-02-27

**Authors:** Sébastien Emery, Luuk Jacobs, Jacob Malich, Gloria Wolkerstorfer, Yiming Dong, Ece Ercan, Jouke Smink, Martijn Nagtegaal, Sebastian Kozerke

**Affiliations:** 1 Institute for Biomedical Engineering, University and ETH Zurich, 8092 Zurich, Switzerland; jacobs@biomed.ee.ethz.ch (L.J.); malich@biomed.ee.ethz.ch (J.M.); wolkerstorfer@biomed.ee.ethz.ch (G.W.); kozerke@biomed.ee.ethz.ch (S.K.); 2 C.J. Gorter MRI Center, Department of Radiology, Leiden University Medical Center, 2333 RC Leiden, The Netherlands; y.dong1@lumc.nl (Y.D.); m.a.nagtegaal@lumc.nl (M.N.); 3 Philips Healthcare, 5684 PC Best, The Netherlands; ece.ercan@philips.com (E.E.); jouke.smink@philips.com (J.S.)

**Keywords:** 4D flow MRI, motion compensation, respiratory binning, self-gating, camera-based binning

## Abstract

Four-dimensional (4D) flow MRI enables the comprehensive assessment of cardiovascular hemodynamics. To compensate for respiratory motion, self-gating strategies are typically used and perform reliably at clinical field strengths. With the recent push towards field strengths below 1 Tesla, these strategies need to be re-evaluated given the reduced signal-to-noise ratio (SNR). Camera-based, contactless respiratory monitoring offers an attractive alternative to self-gating, as it is unaffected by imaging. This study compared respiratory self-gating (SG) and camera-based (VE) binning for phase-contrast gradient-echo (PC-GRE) 4D flow MRI at 0.6 T. Data were acquired from twenty healthy subjects (age: 32.8 ± 12.6 years) using a pseudo-spiral undersampled Cartesian four-point velocity encoding scheme. Reconstructions were performed with FlowMRI-Net for the end-expiratory state using either SG or VE binning. SG and VE showed strong agreement, with cross-correlation coefficients of ~0.87, accuracies of ~0.87, and F1-scores of ~0.9. Velocity analysis revealed high concordance (R^2^ = 0.99; RMSE = 3.9 cm/s), with mean differences in peak velocity of 1.25 ± 2.36 cm/s. In this feasibility study, respiratory self-gating and camera-based binning yielded similar hemodynamic parameters from PC-GRE 4D flow MRI at 0.6 T, with the camera-based approach being independent of MR image SNR.

## 1. Introduction

Four-dimensional (4D) flow magnetic resonance imaging (MRI) is a time-resolved 3D phase-contrast (PC) technique that encodes blood flow velocities in all three spatial directions [1]. It enables comprehensive assessment of cardiovascular hemodynamics, from volumetric flow rates and peak velocities to advanced metrics such as wall shear stress, pressure gradients and 3D flow particle path visualization [2]. However, physiological motion, particularly due to respiration, represents one of the primary sources of artifacts in 4D flow MRI [3]. Consequently, respiratory compensation strategies have become a key objective in the development of 4D flow MRI protocols to enable broader clinical adoption.

Early approaches for respiratory motion compensation in 4D flow MRI relied on prospective gating using bellows [4,5,6] or navigators [7,8,9] to acquire data only during a restricted phase of the respiratory cycle. While these methods improved image quality, they also prolonged scan times and reduced acquisition efficiency, often resulting in clinically impractical examinations [10]. To overcome these limitations, more recent methods acquire data continuously in free breathing and retrospectively sort the measurement data into different respiratory bins using a surrogate signal [11]. Typically, the end-expiratory bin is reconstructed as it is the most reproducible respiratory phase [12]. Surrogate signals may be derived using MR-based self-gating (SG) [13,14,15,16] or other contactless approaches, such as video camera [17,18,19], pilot tone [10,20,21,22,23], or radar [24,25,26] approaches.

MR data-driven binning or self-gating derives the respiratory signal from the periodic sampling of the k-space center (k_0_(t)) [27,28,29] or from a one-dimensional profile (S(x,t)) [13,14,15,30,31,32]. In practice, SG often relies on projections spatially resolved along the superior–inferior (SI) direction, which provide robustness and specificity [33] of the respiratory signal arising from the periodic modulations of the heart–liver and lung–diaphragm interfaces. The breathing motion is typically extracted from the 1D profiles using principal component analysis (PCA). SG techniques have been widely implemented at clinical field strengths (1.5 T and 3 T), particularly for cine [15,28,29,30,34] and for PC imaging [11,13,31,35,36,37,38], where the high contrast-to-noise ratio (CNR) of the anatomical interfaces ensures robust respiratory signal extraction. However, with reduced SNR and CNR at lower magnetic fields, the respiratory modulation may become small relative to other signal components, potentially compromising respiratory signal extraction.

In recent years, mid-field MR systems operating at field strengths between 0.1 and 1 T have regained attention due to advantages such as reduced power deposition, favorable field homogeneity, and overall ease of use [39,40], which could facilitate more widespread adoption of 4D flow MRI in clinical practice. However, the intrinsically lower SNR at these field strengths may compromise SG, making a reassessment necessary. Most of the existing mid-field literature has focused on comparisons using standard clinical protocols, which are predominantly breath-held cine 2D acquisitions [41,42,43]. Recently, Sieber et al. compared a 5D CMR protocol to 2D cine reference scans and showed concordant results [44]. Recent mid-field research on flow imaging has primarily focused on PC balanced steady-state free precession (bSSFP) sequences [27,45] to exploit the higher intrinsic SNR and robust performance of bSSFP at these field strengths, leaving a gap regarding gradient-echo (GRE) 4D flow MRI, where the CNR of the heart–liver and lung–diaphragm interfaces may challenge reliable respiratory signal extraction. To this end, camera-based systems may provide a field strength-independent method for monitoring respiratory motion. The VitalEye (VE) implementation [19], for example, uses a camera to track chest motion to derive a respiratory signal [46,47]. The adoption of camera-based monitoring has been demonstrated across multiple applications, including abdominal imaging [48], cine imaging [49], 4D flow MRI [12,19], lung imaging [50,51], and cholangiography [52,53]. In the context of 4D flow MRI, prior studies have compared camera-based signals to navigators [19] or performed small-scale comparisons among camera, navigator, and self-gating against 2D reference acquisitions [54]. Despite these results, a direct comparison between camera-based and self-gating approaches for 4D flow MRI at mid-field has not yet been performed.

The objective of the present study is to demonstrate PC-GRE 4D flow MRI on a prototype 0.6 T scanner with high-performance gradients and to compare self-gating versus camera-based gating for respiratory motion compensation.

## 2. Methods

### 2.1. Signal Considerations

In radio-frequency-spoiled GRE [55], the transverse steady-state magnetization ($M_{ss}$) is given by:

(1) $$M_{SS}\propto M_{0}\left(B_{0}\right)\frac{\left(1-e^{-TR/T1\left(B_{0}\right)}\right)sin(\alpha )}{\left(1-cos(\alpha )e^{-TR/T1\left(B_{0}\right)}\right)}e^{-TE/{T2}^{*}\left(B_{0}\right)},$$

where $T_{R}$ is the repetition time, $T_{1}$ and ${T_{2}}^{*}$ are the longitudinal and transversal relaxation times, α is the flip angle, $M_{0}$ is the equilibrium magnetization and $B_{0}$ is the field strength.

**Figure 1:**
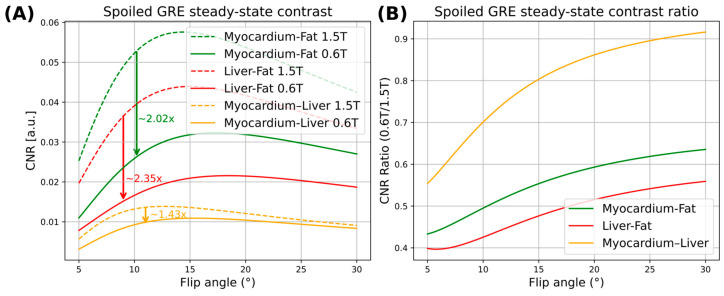
(**A**) Comparison of contrast-to-noise ratios (CNR) for tissue combinations present in the projection at the heart–liver interface as a function of flip angle. Tissue combinations include myocardium (heart)-liver, liver-fat and myocardium-fat. (**B**) Corresponding CNRs (right) for 0.6 T versus 1.5 T.

Respiration-induced motion is reflected by superior–inferior (SI) displacement of the heart–liver interface [33], making this anatomical interface the primary contributor to the motion visible in 1D projection profiles. These profiles also include signal contributions from subcutaneous and abdominal fat. Because fat appears bright in T_1_-weighted imaging, elevated fat signals can partially obscure the modulation arising from the heart–liver interface. A comparison of relative contrast-to-noise ratios (CNR) between 0.6 T and 1.5 T for fat, myocardium, and liver interfaces depending on flip angle is shown in Figure 1. The corresponding ratios between 0.6 T and 1.5 T are included. Relaxation parameters ($T_{1}\;and\;{T_{2}}^{*}$) for 0.6 T and 1.5 T were taken from [39]. Accordingly, a 2.0–2.4 reduction in CNR is expected at 0.6 T when compared to 1.5 T, potentially compromising the accuracy and precision of self-gating approaches.

### 2.2. Self-Gating

**Figure 2:**
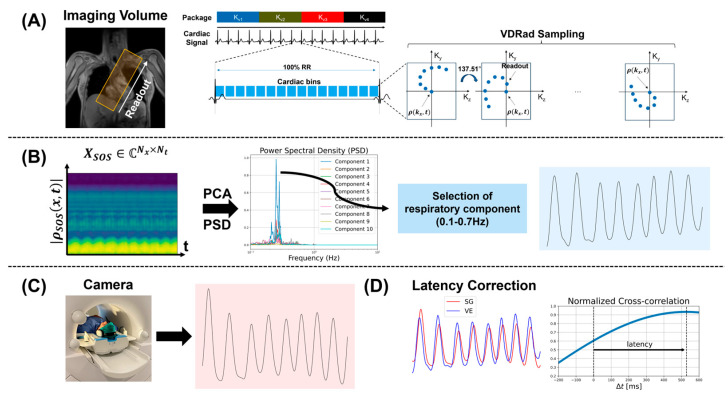
Overview of data acquisition and respiratory signal processing. (**A**) Pseudo-spiral Cartesian sampling scheme (VDRad) with sampling of the center of k-space at each spiral arm ($\rho (k_{x},\;t)$). All data (=package) for one velocity encoding (K_vi_) were collected before acquiring the next velocity encode (K_vi+1_) to reduce eddy current (EC)-related modulations. (**B**) Respiratory motion is extracted from 1D profiles over time, yielding signal modulation at the heart-liver interface. Data from different coils are combined using the sum-of-squares (SOS) method to obtain projection matrix $X_{SOS}(x,\;t)$. Thereafter, principal component analysis (PCA) is applied and the first ten principal components are extracted. The principal component with the maximum power within the breathing frequency range of [0.1–0.7] Hz of the power spectrum density (PSD) is selected as the breathing signal. (**C**) Camera system (VE) to record chest motion of the subject, providing an SNR- and EC-independent measurement of the respiratory signal. (**D**) Example respiratory signals (SG and VE). The normalized cross-correlation is used to correct for the systematic delay between the two signals.

Self-gating signals are obtained by repetitively sampling the k-space center profiles as part of, e.g., pseudo-spiral Cartesian sampling (VDRad) [11] (Figure 2A):

(2) $$S_{c}(k_{x},t)=\int \rho_{c}(x,t)\cdot e^{ik_{x}x}dx,$$

where $S_{c}(k_{x},t)$ is the 1D k-space profile for coil $c(c\in [1,N_{C}])$ at time t $\left(t\in [1,N_{t}]\right)$ and $\rho_{c}\left(x,t\right)$ is the respective coil-weighted profile at that time.

Upon inverse Fourier transform of $S_{c}(k_{x},t)$, the projection matrix $X_{c}(x,t)\in C^{N_{x}\times N_{t}}$ of the excited volume resolved along the spatial axis x over time is obtained. The projection was sampled approximately every 45 ms in our work, leading to a sampling rate of 22 Hz. The magnitude of the signal was used to derive the respiratory signal.

To extract respiratory motion from multi-coil 1D profiles, several strategies have been proposed in the literature [30,56]. Owing to spatially varying coil sensitivity profiles, individual coils emphasize different anatomical regions within the field of view. In this study, coil information was combined using a sum-of-squares (SOS) approach [28] (Figure 2B):

(3) $$X_{sos}=\sqrt{\sum_{c=1}^{N_{c}}{\left|X_{c}\right|}^{2}}\in R^{N_{x}\times N_{t}},$$

and principal component analysis (PCA) was applied to the resulting SOS projection. The first ten principal components were computed, and the respiratory motion signal was identified as the component exhibiting the maximum spectral power density within the expected breathing frequency range [0.1–0.7] Hz.

All data (=package) for one velocity encode (K_vi_) were collected before acquiring the next velocity encode (K_vi+1_) to reduce eddy current (EC)-related modulations of the SG signal [11] (Figure 2A).

### 2.3. Camera Gating

The camera was positioned to target the subject’s chest, capturing movements associated with breathing (Figure 2C). The recorded video was divided into equally sized rectangular patches, and video patches exhibiting periodic respiratory motion were automatically identified [47]. These patches were then weighted according to the strength of the detected motion, and the resulting signal was combined to provide an estimate of the subject’s respiratory motion.

To address potential processing and physiology-related latency of the camera-based respiratory signal [54], the VE signal y(t) was temporally aligned to the SG signal x(t)  using normalized cross-correlation (Figure 2D). The relative delay Δt was determined using:

(4) $$\underset{\Delta t}{\max}\frac{cov(x(t),y(t+\Delta t))}{\sqrt{var(x(t))var(y(t+\Delta t))}}$$

### 2.4. Data Acquisition and Reconstruction

Pseudo-spiral undersampled Cartesian four-point 4D flow MRI [11] was implemented, and data were acquired on a prototype 0.6 T MRI system (Philips Healthcare, Best, The Netherlands) equipped with high-performance gradients (max strength: 45 mT/m, max slew rate: 200 T/m/s) and a 32-channel receive coil. Data were obtained from 20 volunteers (age: 32.8 ± 12.6 years, Table 1) following written informed consent and in compliance with institutional and ethical guidelines.

All scans were retrospectively cardiac-gated using a vector electrocardiogram (VCG), and the camera-based respiratory signal was obtained using the VitalEye (VE) camera [19]. A sagittal oblique field-of-view (360 mm × 240–188 mm × 60 mm) covering the thoracic aorta was prescribed (Figure 2A), with an isotropic spatial resolution of 2.5 mm^3^. Further parameters are listed in Table 2. Additionally, three separate two-point breath-hold 2D PC-GRE scans, encoding velocities in- and through-plane, were acquired as reference, using a transverse slice positioned perpendicular to the ascending aorta at the level of the pulmonary trunk (Table 2).

**Figure 3:**
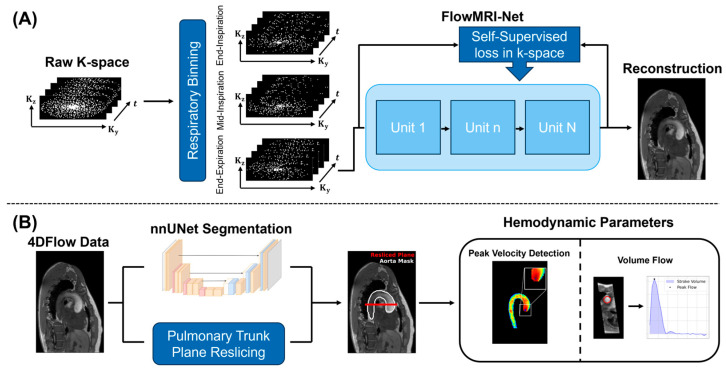
(**A**) Data reconstruction pipeline. The acquired k-space data are binned into three equally populated respiratory bins using either the SG or VE signals. The end-expiratory state is reconstructed using FlowMRI-Net [12]. (**B**) The reconstructed 4D Flow MRI data are processed using an automated aortic vessel segmentation network (nnUNet), generating aortic masks (white). Subsequently, a 2D axial slice is extracted at the level of the pulmonary trunk (red) and peak velocities, volume flow and stroke volume are compared relative to the 2D flow MRI reference and between the 4D Flow MRI reconstruction using either SG or VE for respiratory binning.

Respiratory gating was performed using either the SG signal or the VE system to bin the k-space data into three equally populated respiratory bins (Figure 3A). This binning strategy was chosen to provide a compromise between residual intra-bin respiratory motion and the increase in the effective undersampling associated with subdividing the data. Reconstruction was subsequently performed for the end-expiratory state (Figure 3A), resulting in an effective undersampling factor of approximately 12–13.5 for the end-expiratory bin.

Data using either SG or VE binning were reconstructed using FlowMRI-Net [12] (Figure 3A). Transfer learning was employed by initializing FlowMRI-Net with publicly available weights from pre-training on ten aortic 4D flow MRI acquisitions acquired at 1.5 T [57], which were subsequently fine-tuned for 100 epochs on four held-out acquisitions of the current 0.6 T data. Fine-tuning took ~2 days on an NVIDIA Titan RTX GPU and 32-core Intel Xeon Gold 6130 CPU. Once trained, image reconstruction took ~3 min per subject. Reconstructions were corrected for concomitant field terms [58] and first-order background phase correction was applied [59]. Potential velocity aliasing in-plane (AP and RL) was corrected using a 4D Laplacian algorithm [60].

The 2D PC-GRE scans were reconstructed using a standard parallel-imaging reconstruction pipeline available in precon (GyroTools LLC, Winterthur, Switzerland).

### 2.5. Data Analysis

#### 2.5.1. Respiratory Analysis

To compare respiratory curves derived using SG (x(t)) and VE (y(t)), the maximum of the cross-correlation function:

(5) $$\rho_{xy}(\tau )=\frac{cov(x(t),y(t+\tau ))}{\sigma_{x}\sigma_{y}}$$

was quantified, where $\sigma_{x}$ and $\sigma_{y}$ denote the standard deviations of x(t) and y(t), and cov(x(t),y(t)) denotes the covariance between the two signals.

To evaluate bin-level agreement, the end-expiratory state was treated as the positive class and the other two bins as the negative class, effectively formulating the problem as a binary classification task. The binning accuracy was computed as the fraction of time points for which the two signals were assigned to the same respiratory bin:

(6) $$accuracy=\frac{TP+TN}{TP+TN+FP+FN}$$

where TP are the true positives, TN the true negatives, FP the false positives and FN the false negatives. A respiratory consistency matrix was generated to provide characterization of bin-level agreement and disagreement, while the F1-score was calculated as a metric that simultaneously accounts for both precision and recall:

(7) $$F_{1}=\frac{2\times recall\times precision}{recall+precision}=\frac{2TP}{2TP+FP+FN}$$

#### 2.5.2. 2D Analysis

An axial 2D slice orthogonal to the ascending aorta was extracted from the 3D volume at the level of the pulmonary trunk to enable comparison of flow rate and stroke volume between 2D and 4D flow MRI data (Figure 3B). Correlation plots and Bland–Altman analyses between VE and SG relative to the 2D reference are provided. The statistical significance of differences was calculated using a paired Wilcoxon signed-rank test with *p* < 0.05.

#### 2.5.3. 3D Analysis

To compare 3D velocity vector field maps between SG and VE, the aorta was automatically segmented using a customized nnUNet network [61,62] (Figure 3B). The velocity maps were subsequently computed and masked using the segmentation mask (M(x,y,z)). To compare SG and VE-based reconstructions, the root-mean-square error (${RMSE}_{v}$) was computed within the vessel mask:

(8) $${RMSE}_{v}(u,v)=\sqrt{\sum_{i\in M}{\left\|u_{i}-v_{i}\right\|}^{2}}$$

for the vector field components $u_{i}$ (SG) and $v_{i}$ (VE) and averaged across all cardiac phases.

The mean directional error (mDirrErr) within the vessel mask was computed for the systolic frames as:

(9) $$mDirrErr(u,v)=\frac{1}{\left|M\right|}\sum_{i\in M}1-\frac{\langle u_{i},v_{i}\rangle }{{\left\|u_{i}\right\|}^{2}{\left\|v_{i}\right\|}^{2}}.$$

and averaged over these frames.

Furthermore, the velocity magnitude (${vel}_{mag}=\sqrt{{v_{x}}^{2}+{v_{y}}^{2}+{v_{z}}^{2}}$) was computed and a pixelwise correlation analysis was performed. The coefficient of variation ($R^{2}$) is reported as a measure of the goodness of fit.

Peak velocities within the ascending aorta were extracted (Figure 3B), and agreement between VE and SG was assessed using Bland–Altmann analysis. To identify the region of maximum velocities, a non-maximum suppression strategy was employed at peak systole. First, a maximum intensity projection (MIP) was computed across the slice dimension to generate a 2D MIP map:

(10) $${vel}_{mip}(x,y)=\underset{z\in Z:M(x,y,z)=1}{\max}{vel}_{mag}(x,y,z).$$

Subsequently, a sliding-kernel analysis was performed within the aortic mask. For each kernel position, the mean velocity was calculated, and the kernel yielding the highest mean velocity was designated as the peak velocity location:

(11) $$\mu \left(p\right)=\frac{1}{\left|K_{p}\right|}\sum_{\left(i,j\right)\in K_{p}}{vel}_{mip}\left(i,j\right),\;p^{*}=\underset{p}{\max}\mu \left(p\right),$$

where $K_{p}$ is a (4 × 4) kernel with all pixels belonging to M(x,y,z). Peak velocity was then defined as the maximum voxel-wise velocity contained within this kernel:

(12) $${vel}_{peak}=\underset{\left(i,j\right)\in K_{p^{*}}}{\max}{vel}_{mip}(i,j)$$

## 3. Results

### 3.1. Respiratory Gating Analysis

**Figure 4:**
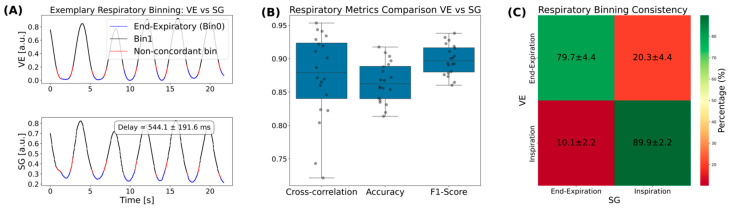
Respiratory gating analysis. (**A**) Example respiratory curves for camera-based binning (VE) (**top**) and self-gating (SG) (**bottom**). The mean delay between the two signals is shown in the bottom plot. (**B**) Boxplots of cross-correlation, binning accuracy, and F1-score across all volunteers. (**C**) Respiratory binning consistency showing correspondence between VE and SG-based binning (mean ± SD, %).

Exemplary VE and SG signals are shown in Figure 4A with indicated respiratory binning. The mean delay between the signals was found to be 544 ms across the cohort. The corresponding cross-correlation was 0.87 ± 0.07, the accuracy 0.87 ± 0.03 and the F1-score 0.90 ± 0.02 (Figure 4B). Respiratory consistency analysis (Figure 4C) revealed that 79.7 ± 4.4% of bins corresponding to the end-expiratory state were concordant between both respiratory gating methods and that 20.3 ± 4.4% were non-concordant. The inspiration bin concordance was 89.9 ± 2.2% (10.1 ± 2.2% discordant).

### 3.2. 2D Analysis

**Figure 5:**
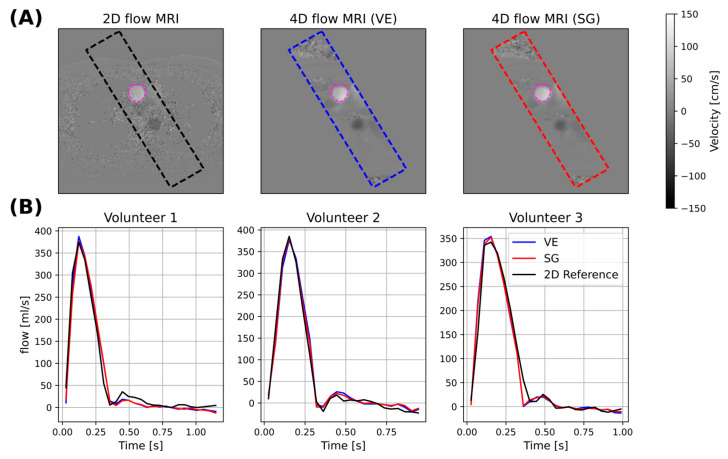
(**A**) Representative exemplary through-plane velocity maps of the 2D flow MRI reference (**left**) relative to maps from corresponding slices extracted from the 4D flow MRI data using VE and SG (**middle**, **right**). (**B**) Comparison of time-resolved through-plane aortic flow in three subjects.

An example peak-systolic through-plane velocity map of the 2D flow MRI reference is compared to corresponding planes extracted from VE and SG 4D flow MRI data in Figure 5A.

Figure 5B shows the extracted flow curves from the 2D reference and the resliced 2D plane from VE and SG for three different volunteers. The flow profiles agree qualitatively for the three different cases.

**Figure 6:**
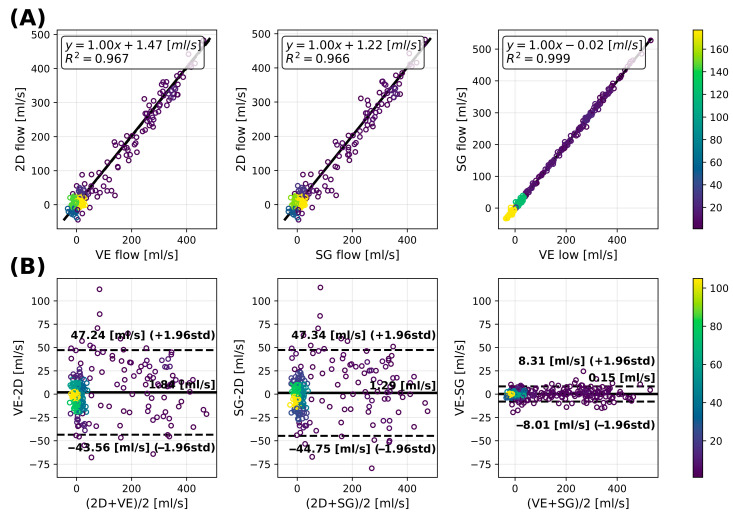
(**A**) Flow correlation analysis between 2D and 4D VE (**left**), 2D and 4D SG (**middle**) and 4D VE and 4D SG (**right**) and (**B**) Bland–Altman analysis (**A**). The color bar indicates the number of touching circles.

Quantitative results of the flow analysis are presented in Figure 6 and evaluated using correlation and Bland–Altman analyses. Good correlations were observed between the 2D flow and the 4D flow acquisition for both VE and SG (Figure 6A; R^2^ = 0.967 and R^2^ = 0.966, respectively). An excellent correlation was also observed between the VE and SG methods (R^2^ = 0.999). Bland–Altman analysis (Figure 6B) demonstrated a small bias between the 2D reference and the VE and SG methods (1.84 and 1.29 mL/s, respectively), with limits of agreement (−43.56 to 47.24 mL/s and −44.75 to 47.34 mL/s, respectively). In contrast, no relevant bias was observed between the VE and SG methods (0.15 mL/s), with narrow limits of agreement (−8.31 to 8.01 mL/s).

**Figure 7:**
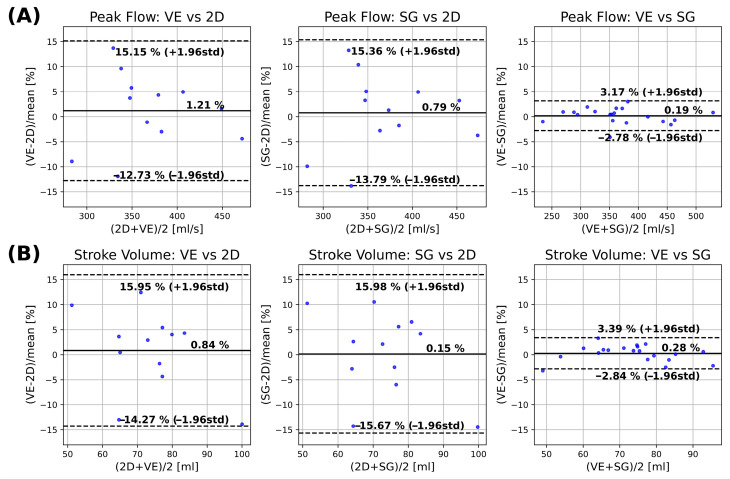
Bland–Altman analysis of peak flow (**A**) and stroke volume (**B**) between 2D and 4D VE (**left**), 2D and 4D SG (**middle**) and 4D VE and 4D SG (**right**).

Agreement of hemodynamic parameters is shown in Figure 7 for peak flow (Figure 7A) and stroke volume (Figure 7B). Peak flow analysis shows a small bias between 2D flow and 4D flow MRI for VE and SG (1.21% and 0.79%), neither of which was significantly different from zero (*p* = 0.582 and *p* = 0.622). The corresponding limits of agreement were (−12.73 to 15.15% and −13.79 to 15.36%). Comparison between 4D VE and SG shows a non-significant bias (0.19%, *p* = 0.43) with narrow limits of agreement (−2.78 to 3.17%). Stroke volume analysis revealed biases between the 2D reference and the 4D VE and SG methods (0.84% and 0.15%, respectively), neither of which were statistically significant (*p* = 0.622 and *p* = 0.850). The corresponding limits of agreement were (−14.27 to 15.95% and −15.67 to 15.98%). Comparison between the 4D VE and SG methods showed a non-significant bias (0.28%, *p* = 0.368) with limits of agreement of (−2.84 to 3.39%).

### 3.3. 3D Analysis

**Figure 8:**
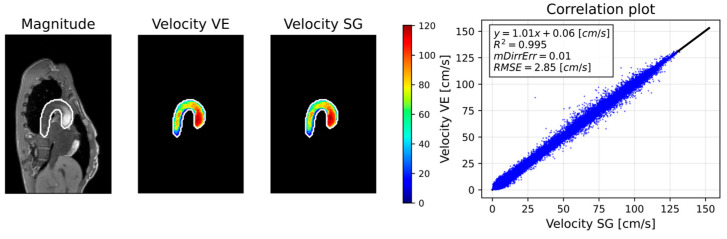
Exemplary 3D velocity field analysis for one volunteer. The first column shows reconstructed magnitude images with the segmented aortic contour (white). The second and third columns display segmented velocity magnitudes for VE- and SG-based reconstructions, respectively. The right column shows the correlation analysis between both methods (R^2^). Pixelwise velocity metrics (RMSE, mDirErr) within the segmented aorta are reported.

Figure 8 illustrates 4D flow reconstructions using VE versus SG in one volunteer, along with corresponding velocity correlations. Table 3 summarizes the results of the velocity field analysis across the cohort. A linear relationship was measured across all volunteers, with an average $R^{2}=0.99\pm 0.009$ and an intercept of 0.19 ± 0.28 cm/s. The velocity RMSE was 3.9±1.02 cm/s, and the directional error (mDirErr) was 0.01±0.007.

**Figure 9:**
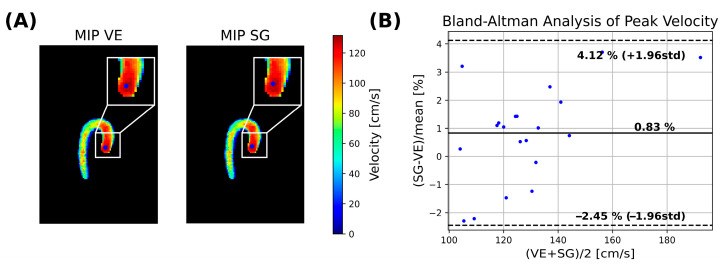
(**A**) Maximum intensity projection (MIP)-based extraction of peak velocity within the ascending aorta for one volunteer. The MIPs are shown for VE (**left**)- and SG (**right**)-based reconstructions, with peak velocity highlighted with a blue dot. (**B**) Bland–Altman analysis of extracted peak velocities across the volunteer cohort.

Peak velocity comparison (Figure 9B) yielded a bias of −0.83% (*p* = 0.064), with limits of agreement of (−2.45% to 4.12%).

## 4. Discussion

4D flow MRI was successfully implemented on a prototype 0.6 T system. Good agreement between the VE and SG signals was found after delay correction, indicating that binning into the end-expiratory bin using either VE or SG was sufficiently reliable. Overall, the analysis of respiratory binning demonstrated a good correlation, high accuracy, and high F1-score between VE and SG. Bin assignment was concordant in about 80% of cases between VE and SG. The remaining 20% is attributed to differences in signal amplitude and shape between VE and SG signals, given that different body structures are tracked with the two approaches.

Compared with the 2D flow MRI reference, 4D flow MRI showed a tendency toward slight overestimation of flow for both VE and SG. The variability of both 4D flow methods compared with the 2D reference was similar. Subsequent direct comparison between VE and SG further demonstrated that the two methods were in close agreement, with negligible bias and low variability for both flow and derived hemodynamic parameters.

The slight overestimation of 4D flow relative to the 2D reference is somewhat counterintuitive and contrasts with the general expectations described in the 4D flow consensus paper [2]. In this study, a slice thickness of 10 mm was employed to increase the SNR of 2D flow MRI at 0.6 T, making the measurements more susceptible to misalignment and partial-volume effects [63]. Another contributor may relate to the velocity encoding package interleaving scheme, which increases the sensitivity to hemodynamic changes over the duration of the scan compared with TR- or beat-interleaving schemes. Further studies are needed to investigate these effects.

Comparison of velocity flow fields revealed good agreement between VE and SG reconstructions. Vector field fidelity, evaluated using the root-mean-square error (RMSE) of velocity vectors, demonstrated low error levels, while directional errors calculated over systolic frames were slightly lower than values previously reported in the literature [12]. Peak velocity estimates further exhibited good agreement, with SG- and VE-based reconstructions showing negligible bias and narrow limits of agreement in Bland–Altman analysis.

This study has several limitations. First, the relatively small number of volunteers and hence limited statistical power prevents drawing generalizable conclusions regarding the comparison between the two gating methods. Second, the study population consisted of relatively lean volunteers only. Therefore, future work should evaluate the robustness of both SG and VE across broader body morphologies and in larger cohorts. Third, the SG and VE signals were aligned using cross-correlation to account for temporal delays between the two signals. This approach required the presence of both signals and cannot be applied when only VE is available. For the purposes of this feasibility study, we applied the alignment, but would like to note that further development of delay correction strategies is required in the future.

## 5. Conclusions

In this feasibility study, respiratory self-gating and camera-based respiratory binning yielded similar image quality and hemodynamic measurements from 4D flow MRI at 0.6 T. The camera-based approach offers practical advantages, as it is independent of imaging SNR. Further studies in larger and more diverse populations are needed to fully assess the robustness and generalizability of the methods at mid-field.

## Figures and Tables

**Table 1:** Characteristics of the volunteer cohort.

Characteristic	Value
Number of Volunteers	20
Age [years]	32.8 ± 12.6
Heart Rate [bpm]	59.5 ± 8.3
Weight [kg]	67.2 ± 7.3
Height [cm]	174.4 ± 5.1
Females (%)	50

**Table 2:** Protocol parameters of the 2D and 4D Flow MRI scans.

Parameters	2D Flow	4D Flow
ECG gating	Retrospective	Retrospective
Respiratory compensation	Breath-hold	SG or VE Gating
FOV [mm^3^]	236–260 × 260–280 × 10	360 × 240–288 × 60
Voxel size [mm^3^]	1.75 × 1.75 × 10.0	2.5 × 2.5 × 2.5
Undersampling factor R	2	~4–4.5
TR [ms]	5.9	5.5–5.9
TE [ms]	3.5	3.3–3.5
Temporal resolution [ms]	41–42	42–46
Flip angle [°]	15	15
V_enc_ [cm/s] (FH-AP-RL)	150–100–100	150–100–100
Bandwidth [Hz]	335	478
Scan duration	~20 s	~9.6 min

**Table 3:** Metrics resulting from the 3D velocity field analysis across all volunteers.

Metrics	Mean ± Std.
RMSE [cm/s]	3.9 ± 1.02
mDirrErr [a.u.]	0.01 ± 0.007
Slope [a.u.]	1.0 ± 0.008
Intercept [cm/s]	0.19 ± 0.28
R^2^ [a.u.]	0.99 ± 0.009

## Data Availability

The original contributions presented in this study are included in the article. Further inquiries can be directed to the corresponding author.

## Abbreviations

The following abbreviations are used in this manuscript:

1D	One-dimensional
3D	Three-dimensional
4D	Four-dimensional
5D	Five-dimensional
RF	radiofrequency
MRI	Magnetic resonance imaging
VE	VitalEye
SG	Self-gating
EC	Eddy currents
PCA	Principal component analysis
SNR	Signal-to-noise ratio
CNR	Contrast-to-noise ratio
BMI	Body mass index
TR	Repetition time
TE	Echo time
SI	Superior-inferior
SOS	Sum-of-square
ECG	Electrocardiogram
FH	Feet-head
AP	Antero-posterior
RL	Right-left
VNR	Velocity-to-noise ratio
LoA	Limits of agreement
VDRad	Variable-Density and Radial view ordering
mDirrErr	Directional error
RMSE	Root-mean-square error
CMR	Cardiovascular magnetic resonance
bSSFP	Balanced steady-state free precession
PC	Phase-contrast
